# *Helicobacter suis* Causes Severe Gastric Pathology in Mouse and Mongolian Gerbil Models of Human Gastric Disease

**DOI:** 10.1371/journal.pone.0014083

**Published:** 2010-11-22

**Authors:** Bram Flahou, Freddy Haesebrouck, Frank Pasmans, Katharina D'Herde, Ann Driessen, Kim Van Deun, Annemieke Smet, Luc Duchateau, Koen Chiers, Richard Ducatelle

**Affiliations:** 1 Department of Pathology, Bacteriology and Avian Diseases, Faculty of Veterinary Medicine, Ghent University, Merelbeke, Belgium; 2 Department of Basic Medical Sciences, Faculty of Medicine and Health Sciences, Ghent University, Ghent, Belgium; 3 Department of Pathology, Maastricht University Medical Centre, Maastricht, The Netherlands; 4 Department of Comparative Physiology and Biometrics, Faculty of Veterinary Medicine, Ghent University, Merelbeke, Belgium; Charité-Universitätsmedizin Berlin, Germany

## Abstract

**Background:**

“*Helicobacter (H.) heilmannii”* type 1 is the most prevalent gastric non-*H. pylori Helicobacter* species in humans suffering from gastric disease. It has been shown to be identical to *H. suis*, a bacterium which is mainly associated with pigs. To obtain better insights into the long-term pathogenesis of infections with this micro-organism, experimental infections were carried out in different rodent models.

**Methodology/Principal Findings:**

Mongolian gerbils and mice of two strains (BALB/c and C57BL/6) were infected with *H. suis* and sacrificed at 3 weeks, 9 weeks and 8 months after infection. Gastric tissue samples were collected for PCR analysis, histological and ultrastructural examination. In gerbils, bacteria mainly colonized the antrum and a narrow zone in the fundus near the forestomach/stomach transition zone. In both mice strains, bacteria colonized the entire glandular stomach. Colonization with *H. suis* was associated with necrosis of parietal cells in all three animal strains. From 9 weeks after infection onwards, an increased proliferation rate of mucosal epithelial cells was detected in the stomach regions colonized with *H. suis*. Most gerbils showed a marked lymphocytic infiltration in the antrum and in the forestomach/stomach transition zone, becoming more pronounced in the course of time. At 8 months post infection, severe destruction of the normal antral architecture at the inflamed sites and development of mucosa-associated lymphoid tissue (MALT) lymphoma-like lesions were observed in some gerbils. In mice, the inflammatory response was less pronounced than in gerbils, consisting mainly of mononuclear cell infiltration and being most severe in the fundus.

**Conclusions/Significance:**

*H. suis* causes death of parietal cells, epithelial cell hyperproliferation and severe inflammation in mice and Mongolian gerbil models of human gastric disease. Moreover, MALT lymphoma-like lesions were induced in *H. suis*-infected Mongolian gerbils. Therefore, the possible involvement of this micro-organism in human gastric disease should not be neglected.

## Introduction

Although infection with *Helicobacter (H.) pylori* is considered to be the major cause of gastritis, peptic ulcer disease [1], [2], gastric adenocarcinoma [3] and mucosa-associated lymphoid tissue (MALT) lymphoma [4] in humans, these gastric diseases have also been associated with other helicobacters, nowadays referred to as gastric non-*H. pylori Helicobacter* (NHPH) species or “*H. heilmannii”* [5]–[8]. The latter, however, has never been a validated species name, since “*H. heilmannii”* represents a group of closely related, but distinct bacterial species, mainly present in different animal species and including *H. felis, H. bizzozeronii, H. salomonis, “Candidatus* H. heilmannii” and *H. suis* [9]. A common feature of these bacteria is their very fastidious nature, which seriously hampers the progress of gastric NHPH-related research. *H. suis* has only recently been cultured *in vitro* [10] and is in fact identical to *“H. heilmannii”* type 1 [11]. It is the most prevalent gastric NHPH species in humans [12], [13]. Moreover, its prevalence is probably underestimated since histological examination of a gastric biopsy, which is commonly performed in humans suffering from gastric disease, is considered not to be the best diagnostic test for infections with *H. suis* and other NHPH species [14].

Numerous studies have boosted the knowledge regarding the pathogenesis of *H. pylori* infections in humans. In contrast, only very few data are available dealing with the pathogenesis of *H. suis* infections in humans [9]. In the past, several infection studies have been performed in mice with *“H. heilmannii”* or “tightly coiled spiral bacteria”, however often without a clear identification of these bacteria to the species level. Moreover, mucus or homogenized gastric tissue of infected mice, pigs or non-human primates was always used as inoculum [15]–[22]. This implies that other micro-organisms were also inoculated along with the helicobacters, which might influence the results, as has been shown for gastric yeasts interfering with a gastric *H. suis* infection in Mongolian gerbils [23].

To obtain better insights into the pathogenesis of human gastric diseases associated with *H. suis*, experimental infections with pure cultures of the bacterium are essential. In *H. pylori* research, different rodent models have been shown to be very useful to obtain significant insights into the pathogenesis of this infection [24], [25]. Therefore, in the present study, C57BL/6 mice, BALB/c mice and Mongolian gerbils were used to explore the interactions between *H. suis* and the gastric mucosa. Mainly in Mongolian gerbils, long-term infection with *H. suis* was associated with severe gastric pathology, including necrosis of gastric epithelial cells and the development of gastric MALT lymphoma-like lesions.

## Materials and Methods

### Ethics statement

The *in vivo* experimental protocol was approved by the Ethical Committee of the Faculty of Veterinary Medicine, Ghent University, Belgium (EC 2007/022; May 21, 2007).

### Animal and bacterial strains

Twenty-seven specific-pathogen-free (SPF) female six-week-old mice of each of two strains (BALB/c and C57BL/6) were purchased from Harlan NL (Horst, The Netherlands). Twenty-seven female SPF outbred gerbils (Crl:MON) of six weeks old were obtained from Charles River Laboratories (Brussels, Belgium). All animals were fed and housed as described previously [26].

*H. suis* strain HS5 was isolated from the gastric mucosa of a sow as described previously [10]. Bacteria were grown under microaerobic conditions (85% N_2_, 10% CO_2_, 5% O_2_; 37°C; 72 h) on biphasic Brucella (Oxoid, Basingstoke, UK) culture plates supplemented with 20% fetal calf serum (HyClone, Logan, UT, USA) and Vitox supplement (Oxoid) [23]. The bacteria were harvested and the final concentration was adjusted to 2×10^8^ viable bacteria/ml, as determined by counting in an improved Neubauer counting chamber.

### Experimental procedure

Both for the mice strains and gerbils, 18 animals were inoculated three times at 48 hours intervals with 0.4 ml of the bacterial suspension. Nine animals of each strain (BALB/c, C57BL/6 and Mongolian gerbil) were inoculated with Brucella broth (Oxoid) with a pH of 5 and served as negative controls. Inoculation was performed intragastrically under isoflurane anaesthesia, using a ball-tipped gavage needle. At 3 weeks, 9 weeks and 8 months after the first inoculation, six *H. suis* infected and three control animals of each group were euthanized by cervical dislocation under isoflurane anaesthesia. The stomach of each animal was resected and samples were taken for PCR analysis, histopathological and ultrastructural examination.

### PCR analysis

From each animal, one sample of approximately 4 mm^2^ was taken both in fundus and antrum. The DNeasy Tissue Kit (Qiagen, Hilden, Germany) was used for DNA extraction according to the manufacturer's protocol. All samples were screened for the presence of *H. suis* DNA using an *H. suis* specific PCR [27].

### Histological and ultrastructural examination

Two longitudinal strips of gastric tissue were cut from the oesophagus to the duodenum along the greater curvature. One strip was fixed in 4% phosphate buffered formaldehyde, processed by standard methods, and embedded in paraffin for light microscopy. Nine consecutive sections of 5 µm were cut. After deparaffinization and hydration, heat-induced antigen retrieval was performed in citrate buffer (pH 6.0) using a microwave oven. Slides were incubated with 3% H_2_O_2_ in methanol (5 min) and 30% goat serum (30 min) to block endogenous peroxidase activity and non-specific reactions, respectively.

On the first section, the *H. suis* colonization density was scored, as shown in Table 1, according to the Updated Sydney System [28] after immunohistochemical staining using a polyclonal genus-specific rabbit anti-*H. pylori* antibody (1/320; DakoCytomation, Glostrup, Denmark) [27]. The second section was stained with haematoxylin and eosin (H&E) to score the intensity of the overall gastritis (infiltration with mononuclear and polymorphonuclear cells), also using a visual analog scale similar to the Updated Sydney System [28] but with some modifications, as shown in Table 2. Both diffuse infiltration and the presence of lymphoid aggregates and lymphoid follicles were taken into consideration. Moreover, these H&E stained sections were used for counting the numbers of neutrophils. For differentiation between T and B lymphocytes, staining of CD3 and CD20 antigens, respectively, was performed on sections three and four, using a polyclonal rabbit anti-CD3 antibody (1/100; DakoCytomation) and a polyclonal rabbit anti-CD20 antibody (1/100; Thermo Scientific, Fremont, USA), respectively. Incubation with primary antibodies directed against *Helicobacter*, CD3 and CD20 was followed by incubation with a biotinylated goat anti-rabbit IgG antibody (1/500; DakoCytomation). After rinsing, the sections were incubated with a streptavidin-biotin-HRP complex (DakoCytomation) and the colour was developed with diaminobenzidine tetrahydrochloride (DAB) and H_2_O_2_. A primary antibody directed against the F4/80 surface marker (1/50; Santa Cruz Biotechnology, Inc., Santa Cruz, USA) was used for highlighting mature macrophages. Detection was done using a rat ABC staining system (Santa Cruz Biotechnology, Inc.) Apoptotic cells were identified by immunohistochemical staining on section six using a rabbit polyclonal antibody directed against active caspase-3 and an anti-rabbit HRP-AEC cell and tissue staining kit (R&D Systems, Minneapolis, USA). Replicating cells were identified on section seven using a mouse monoclonal anti-Ki67 antibody (1/25; Novocastra Laboratories Ltd, Newcastle upon Tyne, UK) and a biotinylated goat anti-mouse IgG antibody (1/200; DakoCytomation). Subsequent visualization was done as described for *Helicobacter*, CD3 and CD20 staining. Parietal cells were identified by immunohistochemical staining for the hydrogen potassium ATPase using a mouse monoclonal antibody (1/200; Abcam Ltd, Cambridge, UK) and a biotinylated goat anti-mouse IgG antibody (1/200; DakoCytomation). Subsequent visualization was done as described for *Helicobacter*, CD3 and CD20 staining. Finally, a monoclonal mouse anti-cytokeratin antibody (1/50; DakoCytomation) was used to highlight lymphoepithelial lesions (LEL's). These sections were further processed using an EnVision^TM^+ system for use with mouse primary antibodies (DakoCytomation).

**Table 1:** Gastric *H. suis* colonization in experimentally infected mice and gerbils.

Time pi a	Group	Colonization score fundus b				Colonization score antrum b			
		0 c	1 c	2 c	3 c	0 c	1 c	2 c	3 c
**3 weeks**	**BALB/c**	0	3	3	0	0	1	3	2
	**C57BL/6**	0	0	6	0	0	0	3	3
	**Gerbil**	1	5	0	0	0	2	2	2
**9 weeks**	**BALB/c**	0	4	2	0	1	2	3	0
	**C57BL/6**	0	2	4	0	0	1	4	1
	**Gerbil**	3	3	0	0	0	3	1	2
**8 months**	**BALB/c**	0	6	0	0	2	2	2	0
	**C57BL/6**	0	1	5	0	0	0	3	3
	**Gerbil**	2	4	0	0	0	0	1	5

a pi: post infection.

b Displayed are the numbers of animals out of six animals in each infected group with a specific colonization score.

c 0, no bacteria; 1, mild colonization; 2, moderate colonization; 3, marked colonization.

**Table 2:** Overall gastric inflammation in experimentally infected mice and gerbils.

Time pi a	Group	Inflammation score fundus b					Inflammation score antrum b				
		0 c	1 c	2 c	3 c	4 c	0 c	1 c	2 c	3 c	4 c
**3 weeks**	**BALB/c**	4	2	0	0	0	4	2	0	0	0
	**C57BL/6**	5	1	0	0	0	5	1	0	0	0
	**Gerbil**	5	1	0	0	0	1	1	2	2	0
**9 weeks**	**BALB/c**	0	4	2	0	0	6	0	0	0	0
	**C57BL/6**	5	1	0	0	0	3	3	0	0	0
	**Gerbil**	1	3	2	0	0	0	0	1	4	1
**8 months**	**BALB/c**	0	0	4	2	0	5	1	0	0	0
	**C57BL/6**	1	1	4	0	0	5	1	0	0	0
	**Gerbil**	0	4	2	0	0	0	0	2	1	3

a pi: post infection.

b Displayed are the numbers of animals out of six animals in each infected group with a specific overall inflammation score.

c 0, no infiltration with mononuclear and/or polymorphonuclear cells; 1, mild diffuse infiltration with mononuclear and/or polymorphonuclear cells or the presence of one small (50–200 cells) aggregate of inflammatory cells; 2, moderate diffuse infiltration with mononuclear and/or polymorphonuclear cells and/or the presence of 2–4 inflammatory aggregates; 3, marked diffuse infiltration with mononuclear and/or polymorphonuclear cells and/or the presence of at least five inflammatory aggregates; 4, diffuse infiltration of large regions with large aggregates of mononuclear and/or polymorphonuclear cells.

During each immunohistochemical staining protocol, appropriate washing steps were included. After counterstaining of nuclei with haematoxylin, slides were dehydrated and mounted.

To determine the numbers of cells belonging to defined immune cell populations (T cells, B cells, macrophages and neutrophils) *in situ*, positive cells were counted in five randomly chosen High Power Fields (magnification: ×400), both in fundus and antrum. For each animal, an average of the positive cell count was determined for both stomach regions. For inflammatory aggregates and lymphoid follicles, the average area percentage of T and B cell populations was determined by using Optimas 6.51 image analysis software (Media Cybernetics, Inc., Bethesda, USA). Finally, for each individual animal, these data were translated into the respective percentages of both lymphocytic populations in aggregates and follicles.

The rates of apoptosis and epithelial cell proliferation were also determined by counting the number of active caspase-3 positive and Ki67 positive epithelial cells, respectively, in five randomly chosen High Power Fields at the level of the gastric pits, both in antrum and fundus. For each animal, an average of the positive cell count was determined for both stomach regions.

The second longitudinal strip of tissue was processed for Transmission Electron Microscopy (TEM) as described previously [26]. Semithin sections (2 µm) were stained with toluidine blue to select the most appropriate regions for ultrathin sectioning. Ultrathin sections were stained with uranyl acetate and lead citrate solutions before examining under a Jeol EX II transmission electron microscope at 80 kV.

### Statistical analysis

Colonization and inflammation scores of infected groups of different animal strains were compared pairwise using the Wilcoxon rank sum test, using time as a stratification factor and a Bonferroni adjusted significance level of 0.05/3 = 0.0167. For analysis of defined immune cell populations, data from the three time points post infection were analyzed separately as appropriate, by analysis of variance with a Bonferroni post hoc test or the Wilcoxon rank sum test with Bonferroni adjustment of significance levels for multiple comparisons. The numbers of Ki67 positive and active caspase-3 positive cells were analyzed by analysis of variance with a Bonferroni post hoc test assuming normally distributed error terms.

## Results

### Bacterial colonization

**Figure 1:**
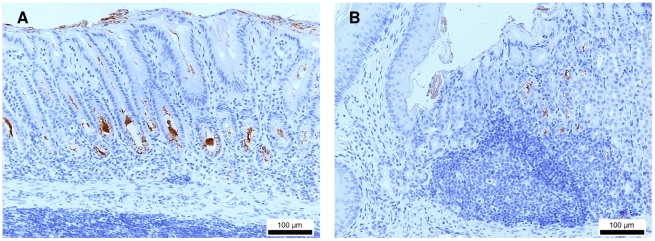
Immunohistochemical *Helicobacter* staining of a gerbil stomach, showing *H. suis* colonization. *H. suis* bacteria (brown) are seen (A) in the glands of the antrum and (B) at the forestomach/stomach transition zone, accompanied by a focal infiltrate of mononuclear cells. Original magnification: 200×.

Throughout the experiment, all control animals were negative for *H. suis* in PCR and immunohistochemical staining. All infected animals were PCR-positive in both antrum and fundus. The colonization levels of the infected animals are shown in Table 1. In general, colonization scores were higher in C57BL/6 mice compared to BALB/c mice (P = 0.0016 for fundus; P = 0.0035 for antrum). For both mice strains and for all timepoints, bacteria were seen throughout the entire glandular stomach, both in antrum and fundus. For the gerbils, however, colonization in the antrum was moderate to marked at all timepoints (Table 1; Figure 1A), whereas colonization in the fundus remained limited to a very narrow zone at the limiting ridge (Figure 1B). Moreover, in some gerbils, no bacteria could be visualized in the fundic region. Although not statistically significant, colonization scores in infected BALB/c mice tended to drop in the course of time (P = 0.18 for fundus; P = 0.08 for antrum), whereas a tendency to increase was observed in the antrum of Mongolian gerbils (P = 0.15).

### Inflammation

At all timepoints, all uninfected control animals showed normal histomorphology, with little inflammatory cell infiltration in the gastric mucosa. For both mice strains and for the gerbils, inflammation in infected animals was characterized by mononuclear and polymorphonuclear cell infiltration in the lamina propria mucosae or the tunica submucosa or both, depending on the individual animal. The individual overall inflammation scores of the *H. suis*-infected animals are shown in Table 2. For BALB/c mice at all three timepoints and C57BL/6 mice at 8 months post infection, inflammation was observed mainly in the fundus. In general, BALB/c mice showed higher inflammation scores in the fundic region, compared to C57BL/6 mice (P = 0.004). In Mongolian gerbils, however,.inflammation in the fundic region was always limited to a very narrow zone near the limiting ridge, i.e. the forestomach-stomach transition zone (Figure 1B). Generally, a more severe inflammation was observed in the antrum of infected gerbils, compared to BALB/c and C57BL/6 mice (P<0.0001).

**Figure 2:**
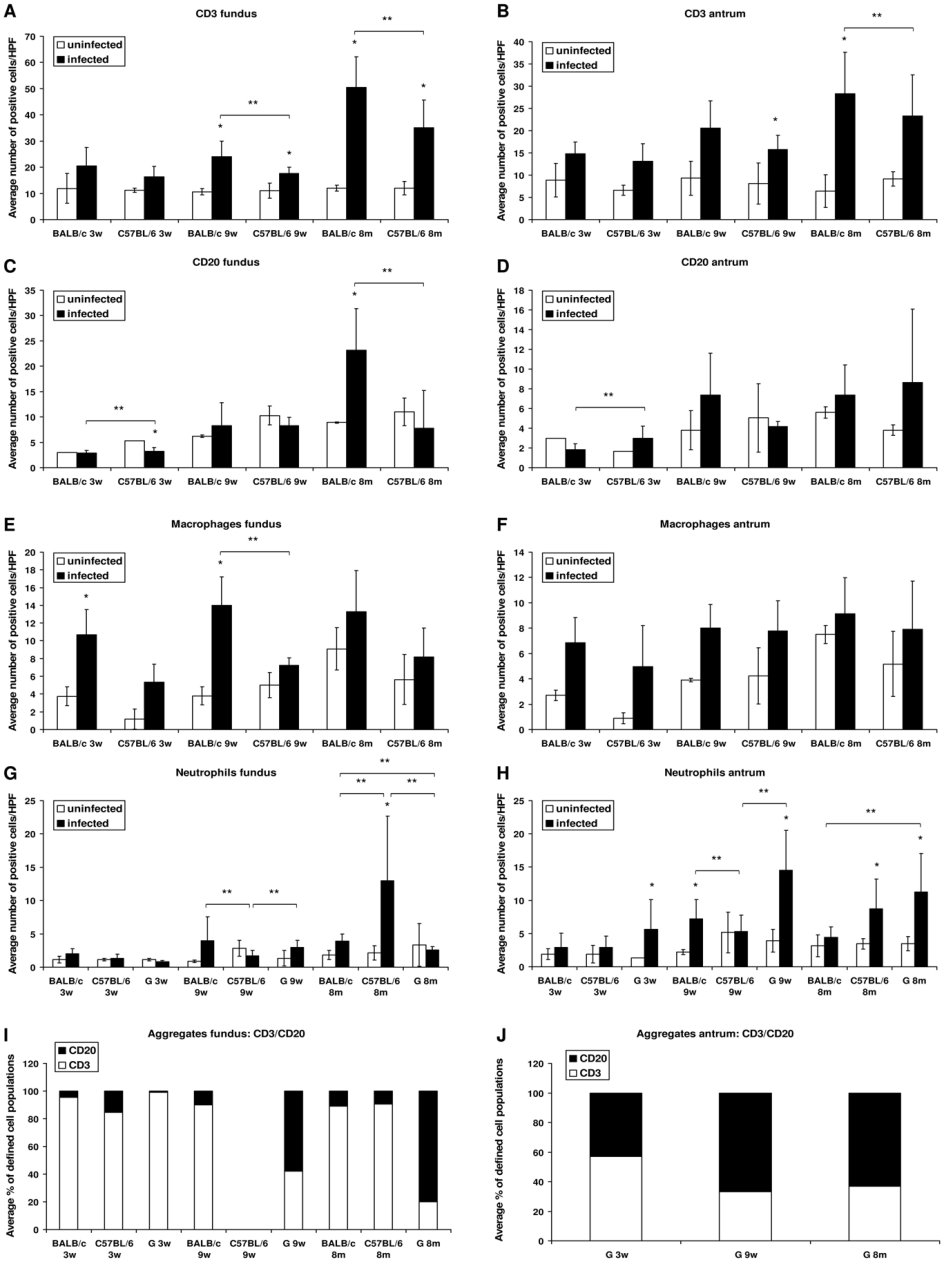
Counting of defined immune cell populations and image analysis of lymphocytic aggregates and lymphoid follicles. (A–H) Shown are the average (±SD) numbers of cells/High Power Field (HPF) belonging to a defined immune cell population, including T cells (CD3-positive), B cells (CD20-positive), macrophages (F4/80-positive) and neutrophils. * depict statistically significant differences between corresponding *H. suis*-infected and uninfected control animals. ** depict statistically significant differences between infected groups of different animal strains at the same time point post infection. (I–J) Shown are the percentages of T (CD3-positive) and B (CD20-positive) lymphocytes in lymphocytic aggregates and lymphoid follicles in *H. suis*-infected animals. BALB/c and C57BL/6, mice strains; G, gerbil; 3w, 3 weeks post infection; 9w, 9 weeks post infection; 8 m, 8 months post infection.

Counting of defined immune cell poulations is shown in Figure 2. At 9 weeks and 8 months post infection, an increase of the number of T cells (CD3-positive) was observed in the fundus of infected animals of both mice strains (Figure 2A). This increase was always higher for BALB/c mice, compared to C57BL/6 mice. Similar results were seen in the antrum, however not always statistically significant (Figure 2B). At 3 and 9 weeks post infection, no statistically significant increase could be observed for diffuse infiltration of the fundic mucosa with B cells (CD20 positive) in infected animals as compared to control animals. However, at 8 months post infection, a higher number of B cells was detected only in the fundus of infected BALB/c mice (Figure 2C). In the antrum of both mice strains, no significant differences were found for B cell infiltration between uninfected and infected animals (Figure 2D). For BALB/c mice infected for 3 and 9 weeks, a significantly higher number of macrophages could be detected in the fundus when compared to the control animals (Figure 2E). For C57BL/6 mice, similar results could be observed, however not statistically significant. Additionally, for the antrum of both mice strains, this increase was never statistically significant (Figure 2F). In both mice strains, neutrophils were always vastly outnumbered by mononuclear cells. At 8 months post infection, infected C57BL/6 mice showed higher numbers of neutrophils, compared to control animals, both in fundus and antrum (Figure 2G and Figure 2H). An increase of the number of neutrophils could be seen in BALB/c mice at 9 weeks post infection, an observation which was absent at the last time point of euthanasia.

**Figure 3:**
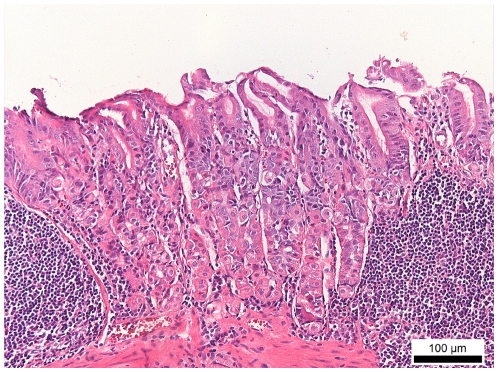
H&E staining of the fundus of a *H. suis*-infected BALB/c mouse. In this animal, infected for 8 months, two large lymphoid aggregates can be seen in the lamina propria mucosae, accompanied by loss of normal mucosal architecture. Original magnification: 200×.

The presence of inflammatory cell aggregates and lymphoid follicles is shown in Table 3. Both the number of *H. suis*-infected animals with inflammatory cell aggregates and the mean number of aggregates/animal were always equal (3 weeks post infection) or higher (9 weeks and 8 months post infection) for BALB/c mice compared to C57BL/6 mice. At 8 months post infection, the fundic region of all *H. suis*-infected BALB/c contained large lymphoid aggregates (Figure 3). For both mice strains, lymphoid aggregates and follicles were mainly composed of T cells (Figure 2I).

**Table 3:** Aggregates of inflammatory cells and lymphoid follicles in mice and gerbils after experimental infection with *H. suis*.

Time pi a	Group	Number of animals b with inflammatory aggregates (>50 cells) c	Mean number of aggregates/animal in this group c	LF e	EGC f
3 weeks	BALB/c	1	0.17	0	0
	C57BL/6	1	0.17	0	0
	Gerbil	NA d	NA d	4	1
9 weeks	BALB/c	5	1.33	1	0
	C57BL/6	0	0	0	0
	Gerbil	NA^d^	NA d	4	4
8 months	BALB/c	6	4.33	2	1
	C57BL/6	4	1.33	1	0
	Gerbil	NA d	NA d	6	6

a pi: post infection.

b out of 6 animals in each infected group.

c both parameters were determined on the entire longitudinal H&E stained tissue sections.

d not applicable.

e presented are the numbers of animals (out of 6 animals in each infected group) with gastric lymphoid follicles (LF).

f presented are the numbers of animals (out of 6 animals in each infected group) with expanding germinal centers (EGC) in gastric lymphoid follicles.

**Figure 4:**
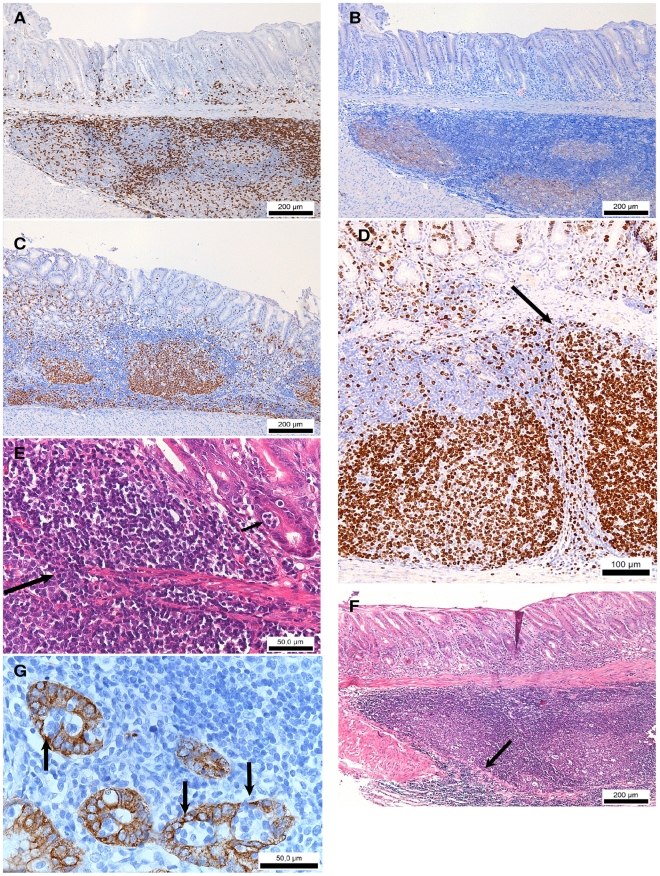
Gastric inflammation in *H. suis*-infected Mongolian gerbils. (A) CD3 staining of the antrum of a gerbil infected with *H. suis* for 8 months, showing T-lymphocytes (brown). Original magnification: 100×. (B) CD20 staining of the antrum of a gerbil infected with *H. suis* for 8 months, showing B lymphocytes (brown) in germinal centers of lymphoid follicles. Original magnification: 100×. (C) Ki67 staining of the antrum of a gerbil infected with *H. suis* for 9 weeks showing distinct proliferation (brown) in the germinal centers of the lymphoid follicles. Original magnification: 100×. (D) Ki67 staining of the antrum of a gerbil infected with *H. suis* for 8 months showing large, hyperproliferative (brown) and irregular (arrow) germinal centers of the lymphoid follicles. Original magnification: 200×. (E) H&E staining of the antrum of a gerbil infected with *H. suis* for 8 months showing lymphocytic infiltration of the lamina muscularis mucosae (large arrow) and a small lymphoepithelial lesion (small arrow). Original magnification: 400×. (F) H&E staining of the antrum of a gerbil infected with *H. suis* for 8 months showing infiltration of the tunica muscularis with mononuclear cells (arrow). Original magnification: 100×. (G) Cytokeratin staining of the antral epithelium (brown) of a gerbil infected with *H. suis* for 8 months showing numerous lymphoepithelial lesions (arrows). Original magnification: 400×.

For *H. suis*-infected Mongolian gerbils, the vast majority of inflammatory cells consisted of T and B lymphocytes. Moreover, diffuse infiltrates and large inflammatory aggregates were very often fused together, which made counting of T and B cell populations virtually impossible. Organization into lymphoid follicles was present in the majority of gerbils at 9 weeks and 8 months post infection (Table 3; Figure 4A and Figure 4B). Image analysis revealed that from 9 weeks of infection onwards, lymphocytic aggregates and follicles in these animals contained at least 60% B cells (CD20 positive and CD3 negative) (Figure 2I and Figure 2J). This fraction was smaller at 3 weeks after infection. In gerbils infected for at least 9 weeks, a distinct B-cell proliferation in the germinal centers of the lymphoid follicles was present (Figure 4C). In gerbils infected for 8 months, these germinal centers were often large, hyperproliferative and irregular (Figure 4D). Moreover, in gerbils infected for 9 weeks and 8 months, severe destruction of the normal antral mucosal architecture with disruption of the lamina muscularis mucosae (Figure 4E) was seen in 4 and in all animals, respectively. In mice, only at 8 months after infection, infiltration and destruction of the lamina muscularis mucosae was detected in 1/3 of the animals. In one gerbil, the tunica muscularis was also invaded by a large number of lymphocytes (Figure 4F). Lymphoepithelial lesions could be detected in the mucosa of 2 gerbils infected with *H. suis* for 8 months (Figure 4E and Figure 4G). At all three timepoints post infection, diffuse infiltration of the antral mucosa with neutrophils was always higher in *H. suis*-infected gerbils compared to control animals (Figure 2H), although this cell type represented only a small fraction of the total population of inflammatory cells. Finally, two primary antibodies (F4/80, Santa Cruz Biotechnology, Inc.; MAC387, Abcam) were used to detect mature macrophages in the gerbils, however without success.

### Gastric epithelial cell death

**Figure 5:**
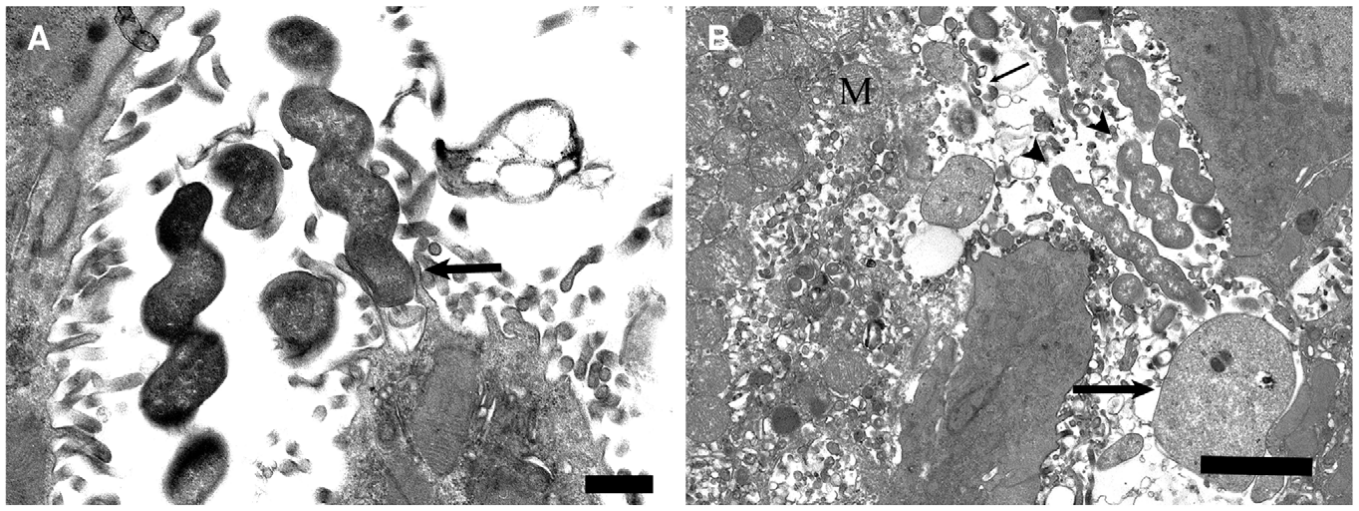
Transmission electron microscopic (TEM) images of BALB/c mice infected with *H. suis*. (A) In a BALB/c mouse infected for 9 weeks, spiral-shaped *H. suis* bacteria are seen in close association with parietal cell microvilli (arrow). Bar = 500 nm. (B) TEM image of a BALB/c mouse infected for 3 weeks with *H. suis* showing *H. suis* bacteria (arrowheads) in between necrotic debris of gastric parietal cells, which can be recognized by the typical abundance of mitochondria (M) and deep folding of the apical membrane. The large arrow indicates the presence of necrotic blebs and the small arrow shows loss of plasma membrane integrity. Bar = 2 µm.

No differences in the average number of apoptotic cells were seen between control and infected animals, for all three animal strains at all timepoints (results not shown). Transmission electron microscopy revealed that spiral-shaped *H. suis* bacteria were often closely associated with gastric epithelial, mainly parietal cells (Figure 5A) in all three animal strains at all three time points post infection. Frequently, these bacteria were seen surrounded by cellular debris of primary necrotic parietal cells with loss of plasma membrane integrity and formation of necrotic blebs (Figure 5B).

**Figure 6:**
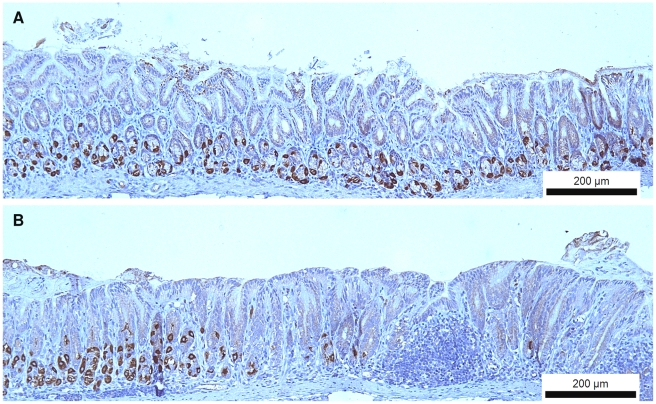
Immunohistochemical staining of the hydrogen potassium ATPase present in gastric parietal cells. (A) Moderate numbers of parietal (brown) cells are still present in the proximal antrum of uninfected Mongolian gerbils. Original magnification: 100×. (B) An abrupt loss of parietal cells is seen in the transition zone between fundus and antrum in Mongolian gerbils infected with *H. suis* for 9 weeks. Original magnification: 100×.

Immunohistochemical staining showed no clear loss of parietal cells in the fundus of infected animals of all three animal strains. However, compared to uninfected animals (Figure 6A), an obvious loss of parietal cells was often detected at the transition zone between fundus and antrum in *H. suis*-infected gerbils at all timepoints post infection (Figure 6B).

### Gastric epithelial cell proliferation

**Figure 7:**
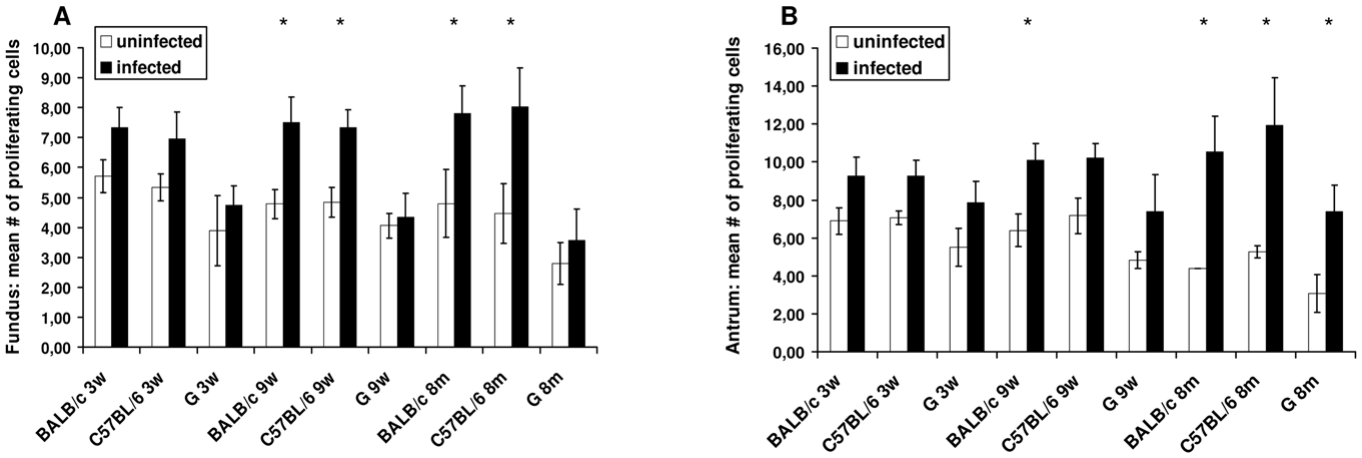
Proliferation of gastric epithelial cells. For each individual animal, the rate of epithelial cell proliferation was determined by counting the number of Ki67 positive epithelial cells in five randomly chosen microscopic fields (magnification: ×400) at the level of the gastric pits, both in antrum and fundus. Shown are the mean numbers of Ki67-positive epithelial cells in each group. An * denotes significantly higher values for *H. suis* infected animals compared to uninfected control animals. BALB/c and C57BL/6, mice strains; G, gerbil; 3w, 3 weeks post infection; 9w, 9 weeks post infection; 8 m, 8 months post infection.

Results of the gastric epithelial cell proliferation scoring are shown in Figure 7. Higher numbers of Ki67-positive, and thus proliferating epithelial cells were seen at 9 weeks and 8 months after infection in both fundus and antrum of *H. suis*-infected BALB/c mice, compared to control animals. Similar results were observed for C57BL/6 mice. In gerbils, a higher proliferation rate was seen in the antrum of infected animals at 8 months after infection. In the fundus of *H. suis*-infected gerbils, no significant increase of epithelial cell proliferation was observed.

## Discussion

In the present study, at 3 and 9 weeks post infection, increased numbers of mature macrophages were observed in the gastric mucosa of *H. suis* infected mice, mainly in the BALB/c strain. In early *H. pylori* infection, macrophages are known to play an essential role as innate responders to released *H. pylori* factors [29], [30]. They have been suggested to activate the adaptive immune response by producing factors such as IL-12, stimulating a Th1 response, and IL-10, which is considered a Th2-polarizing cytokine [30], [31].

At 9 weeks and 8 months post infection, a more pronounced lymphocytic infiltration was observed in fundus and antrum of BALB/c mice, compared to C57BL/6 mice. This contrasts with the results of Cinque et al. [15], who found a higher degree of inflammation in C57BL/6 mice inoculated with gastric mucus from “*H. heilmannii* type 1”-infected pigs, compared to BALB/c mice. Moreover, results similar to those of Cinque et al. [15] have been reported for mice infected with *H. felis* [32]–[34]. This suggests that inflammation in these studies was mainly driven by a T-helper (Th) 1 response, since C57BL/6 mice have been described genetically as Th1 responders [35], [36]. In contrast, BALB/c mice are considered predominant Th2 responders. The higher inflammation in BALB/c mice in the present study might therefore indicate that *H. suis* strain 5 elicits a more predominant Th2 response [24], [35], [36]. Indeed, only in BALB/c mice infected for 8 months, significantly higher levels of B cell accumulation were observed, which suggests a more Th2-polarized response [30], [37]. Although this finding was not reflected in the fraction of B cells in lymphoid aggregates and follicles and although no MALT lymphoma-like lesions were seen in these mice in the present study, this might eventually lead to a severe B-cell lymphocytic proliferation and even gastric MALT lymphoma, as shown for BALB/c mice infected for over 18 months with *H. felis* [38].

Humans suffering from a “*H. heilmannii”* gastritis have been suggested to develop gastric MALT lymphoma more frequently than those suffering from a *H. pylori* gastritis [39]. Interestingly, in the present study, in some gerbils euthanized at 8 months after infection, severe inflammation had evolved to a pathology resembling gastric MALT lymphoma. Similar MALT lymphoma-like lesions have been described in mice infected for at least 18 months with different “*H. heilmannii”*-like strains, isolated *in vivo* in mice [19]. Some of these inocula were shown to contain *H. suis* in a separate paper from the same authors [11]. Nakamura et al. [17] also described the development of gastric MALT lymphoma in C57BL/6 mice infected for at least 6 months with an *in vivo* “isolate” that was erroneously designated “*Candidatus* H. heilmannii”, but that in fact belonged to the species *H. suis* [40]. It is not clear why in the present study MALT lymphoma-like lesions were not detected in the mouse models. Differences in *H. suis* strains and duration of infection probably play an important role [19]. Other micro-organisms which may have been present in the inoculum used by O'Rourke et al. [19] and Nakamura et al. [17] might also have influenced lesion development [23].

In Mongolian gerbils, inflammation was limited to the antrum and a narrow zone at the forestomach-stomach transition zone. These results are not surprising, since *H. suis* mainly colonizes these stomach regions in Mongolian gerbils, while it tends to colonize both antrum and fundus in mice. In “*H. heilmannii”* infected humans, colonization and inflammation are also mainly localized in the antrum [8]. Therefore, the Mongolian gerbil model more closely resembles the human situation. This together with our finding that Mongolian gerbils developed a more severe pathology after *H. suis* infection compared to mice, indicates that the Mongolian gerbil model may be more suitable for studying *H. suis*-host interactions.

In all infected animal strains, a relative absence of neutrophils was observed. Similar findings have been described for mice infected with *H. felis* [32], [35]. However, in the present study, an increase in the number of neutrophils was observed in C57BL/6 mice infected for 8 months. In BALB/c mice, a similar increase was observed, however less pronounced and only at 9 weeks post infection. At 8 months post infection, the level of neutrophil infiltration in this mouse strain had dropped back to basal levels. Possibly, different and fluctuating levels of IL-17 expression, a cytokine which has been shown to be a key regulator of neutrophil infiltration, could play a role, as has been described in long-term *H. pylori* infection studies in mice of different strains [41], [42].

In this study we report that *H. suis* induces necrosis of gastric parietal cells, which may have important implications for the development of various gastric pathologies, such as gastric erosion and/or ulcer formation [43], gastric atrophy and even gastric cancer [44]. These lesions all have been observed in *“H. heilmannii”*-infected humans [5], [6], [8] and are often accompanied by a gastritis [8]. The chronic gastritis observed in the present study is possibly caused by direct effects of *H. suis*, but most likely also driven indirectly by necrosis of gastric epithelial cells. Cell necrosis results in the release of cellular contents, including molecules involved in the promotion of inflammation [45]. Interestingly, mainly parietal cells were affected by necrosis, which has also been described for other non-*H. pylori* helicobacters, such as *H. felis* [26]. In the present study, also a clear increase of gastric epithelial cell proliferation was seen in the *H. suis* infected animals. For *H. pylori*, data derived from *in vitro* experiments suggest that this hyperproliferation may be a secondary response to increased cell death in order to maintain cell mass in the mucosa [44]. In any case, hyperproliferation may result in a shift towards population of gastric epithelium with immature cells, resulting in impaired gastric acid secretion [43]. Both hyperproliferation of the gastric epithelium and reduced gastric acid secretion due to loss of parietal cells may eventually lead to the development of gastric cancer [1], [44].

In conclusion, our results clearly demonstrate the ability of a pure culture of *H. suis* to cause severe gastric pathology. In humans suffering from gastric disease, the possible involvement of this micro-organism should therefore not be neglected.
